# The comparison of censored quantile regression methods in prognosis factors of breast cancer survival

**DOI:** 10.1038/s41598-021-97665-x

**Published:** 2021-09-14

**Authors:** Akram Yazdani, Mehdi Yaseri, Shahpar Haghighat, Ahmad Kaviani, Hojjat Zeraati

**Affiliations:** 1 Department of Biostatistics and Epidemiology, Faculty of Health, Kashan University of Medical Sciences, Kashan, Iran; 2 Department of Epidemiology and Biostatistics, School of Public Health, Tehran University of Medical Sciences, Tehran, Iran; 3 Breast Cancer Research Center, Motamed Cancer Institute, ACECR, Tehran, Iran; 4 Department of Surgery, Tehran University of Medical Sciences, Tehran, Iran

**Keywords:** Cancer, Medical research

## Abstract

The Cox proportional hazards model is a widely used statistical method for the censored data that model the hazard rate rather than survival time. To overcome complexity of interpreting hazard ratio, quantile regression was introduced for censored data with more straightforward interpretation. Different methods for analyzing censored data using quantile regression model, have been introduced. The quantile regression approach models the quantile function of failure time and investigates the covariate effects in different quantiles. In this model, the covariate effects can be changed for patients with different risk and is a flexible model for controlling the heterogeneity of covariate effects. We illustrated and compared five methods in quantile regression for right censored data included Portnoy, Wang and Wang, Bottai and Zhang, Yang and De Backer methods. The comparison was made through the use of these methods in modeling the survival time of breast cancer. According to the results of quantile regression models, tumor grade and stage of the disease were identified as significant factors affecting 20th percentile of survival time. In Bottai and Zhang method, 20th percentile of survival time for a case with higher unit of stage decreased about 14 months and 20th percentile of survival time for a case with higher grade decreased about 13 months. The quantile regression models acted the same to determine prognostic factors of breast cancer survival in most of the time. The estimated coefficients of five methods were close to each other for quantiles lower than 0.1 and they were different from quantiles upper than 0.1.

## Introduction

In many medical studies, the outcome of interest is the time to event. For instance, in cancer, the event of interest is death or the relapse of illness, and in transplantation, the rejection of transplanted organ can be considered as the event. In case of the uncertainty of time for study inclusion and the incidence of an event in some studied units, time is regarded as the censored data^1^. The Cox proportional hazards (Cox) model is a widely used statistical method for the censored data. However, this model is limited by the assumption of a constant hazard ratio (HR) over time (i.e., proportionality), and models the hazard rate rather than the survival time directly^2^. Also, the complexity of the HR estimate interpretation was recognized as a problem in the Cox models. To overcome this limitations, other methods such as accelerated failure time (AFT) models and censored quantile regression (CQR) models were introduced for censored data with a more straightforward interpretation^3^. The AFT model is a model that assumes that a treatment or an exposure either extends or reduces the time to the development of event. Although the AFT model allows a direct interpretation of covariate effects on event time, it requires a strong assumption of homogeneous treatment effect^4^. The AFT model can only capture location shifts effects, and hence may fail to capture heterogeneity of covariate effects. The CQR model does not require the assumption of a homogeneous covariate effect, while the effect estimates have the same straightforward interpretation as those in AFT model^3^. A censored quantile regression model is a model of the quantile function of failure time and investigates the covariate effects in different quantiles. In this model, the covariate effects can be changed for patients with different risks and is a flexible model for controlling the heterogeneity of covariate effects. Further, the failure time can be predicted by CQR model and the progression of a disease can be predicted^4^.

For conditionally random right censoring, various approaches have been proposed that the most practical of them include: Portnoy^5^, Peng and Huang^6^, Wang and Wang^7^, Bottai and Zhang^8^, Yang et al.^9^ and De Backer et al.^10^ methods. Portnoy generalized the Kaplan–Meier method to estimate quantile of survival time with a recursively weighted estimation algorithm under the global linearity assumption of the conditional quantile functions^5^. Peng and Huang presented another method based on Nelson-Aalen estimator of cumulative hazard function requiring linearity assumption like Portnoy’s method and present the result closed to Portnoy method^6^. To overcome the linearity assumption, Wang and Wang developed a method by non-parametrically estimating the conditional survival distribution via kernel smoothing^7^. In 2010, Laplace regression was introduced as a parametric method for modeling the conditional quantile of censored data by Bottai and Zhang^8^. They assumed error term follow asymmetric Laplace distribution and considered the Laplace regression model as a method for modeling the conditional quantiles of survival time. Nevertheless, this parametric assumption, shared by other methods in quantile regression (Liu and Botta^11^, Farcomeni^12^, Lee and Neocleous^13^, Yuan and Yin^14^), has been shown assumption asymmetric Laplace distribution for error term, not to influence the results of the model under different data distributions. It was indicated in simulation studies that correct coverage and shorter computation time obtained with Laplace regression model compared with other alternative methods^8^. Yang et al. to estimate quantiles of survival time, employed a variation of the data augmentation algorithm ^9^. Base on the general principle of data augmentation^15^, in the algorithm, they first, impute censored values from the quantile functions then using the imputed values fit the quantile model. De Backer et al. investigate a new procedure for modeling right-censored data with a linear quantile regression^10^. They used “check” loss function that stems from the influential work of Koenker and Bassett^16^, to circumvent the formulation of conditional quantiles. The assumptions and features of these models are summarized in Table 1.

**Table 1:** Assumptions and features Cox, AFT, Portnoy, Wang and Wang, Bottai and Zhang, Yang and De Backer methods.

models	Assumptions	Advantages	Disadvantages
Cox method	Proportional hazard	No need to consider a specific probability distribution for the survival time; Can used in many types of survival model	The effect of the included covariates is multiplicative The complexity of the HR estimate interpretation
AFT method	The effect of a covariate is to accelerate or decelerate the life course of a disease by some constant Needs homogeneous covariates effect	direct interpretation of covariate effects on event time Can used in many types of survival model	Error term follow a specific probability distribution Failing to capture heterogeneity of covariate effects
Portnoy method	The model at lower quantiles are all linear (global-linearity)	The effect of covariates is not restricted to be constant No distributional assumptions about the regression error term	The 'global' linearity assumption
Bottai and Zhang method	The residuals follow a asymmetric Laplace distribution require -linearity assumption	The effect of covariates is not restricted to be constant Correct coverage and shorter computation time	Error term follow a Laplace distribution
Wang and Wang method	Require a locally linear quantile regression	Not require global-linearity assumption	Requires estimating the true distribution of the outcome variable
Yang method	Operates under the assumption that all the quantile functions are identifiable	Can handle different forms of censoring the estimator can achieve significant efficiency gains over the existing methods	It runs a risk of finding estimates even for non-identifiable quantile functions
De Backer method	Require a locally linear quantile regression	Consistency and asymptotic normality of estimator	Restrict to the estimation of the classical linear regression model

The most of existing methods of estimation for censored quantiles are limited to right censored data. This paper has concentrated on the outcome data that were right censored, as this was the main feature of our motivating dataset. Subsequent work has greatly expanded the applicability of these methods to competing risks^17–19^, recurrent events^20,21^, various censoring types^17,22^ and other settings. For example, Yang et al. proposed a new method for different forms of censoring including doubly censored and interval censored data^9^. Narisetty (2018) introduced a new approach for the cure rate quantile regression model^23^. Chen developed quantile regression estimators and proposed a quantile regression method with time-varying covariates^24^.

Recent years, use of quantile regression has increased in cancer research. Base on PubMed search, there are more than 200 publications on applications of quantile regression related to cancer research from 2015 to 2021. Breast cancer is the most common type of cancer after the lung cancer and the most common cause of death from cancer among women^25^. In 2000, there were 10 million new cases of breast cancer, i.e. 25% of all cancer cases around the world, and, it is expected to reach 15 million in 2025^26^. The five-year survival rate of breast cancer range from less than 40% to 80% in low-income to high-income countries around the world^27^. Knowledge of the survival-associated predictors in breast cancer has an important role in the process of treatment and patient care. Age at diagnosis, tumor size, grade, type of auxiliary treatment (radiotherapy, stage of disease, number of involved lymph nodes, chemotherapy, hormone therapy), type of surgery (Modified radical mastectomy (MRM) and Breast conserving surgery (BCS), metastasis and recurrence have been identified as the most important risk factors of breast cancer survival^28,29^.

The present study aimed to compare quantile regression methods included Portnoy, Wang and Wang, Bottai and Zhang, Yang and De Backer methods with Cox and AFT models. The comparison was made through the use of these methods in modeling the survival time of breast cancer using the data collected from Imam Khomeini hospital, Tehran, Iran.

## Methods

### Data description

The present study aimed to determine the factors affecting breast cancer survival using CQR models. The studied data included 800 females patients with breast cancer (based on breast cancer pathology diagnosis) referring to Imam Khomeini Hospital, Tehran, Iran during 1996–2005. The required data were extracted from patients' files. The latest condition of the patient was informed via phone contact.

This study was approved by the Ethics Committee of School of Public Health & Allied Medical Sciences-Tehran university of Medical Sciences (approval ID: IR.TUMS.SPH.REC.1397.212) and was carried out according to relevant guidelines and regulations. The informed consent was obtained from all participants.

The event of interest was death from breast cancer, and the survival time was defined as the duration (months) from diagnosis to death due to breast cancer. The prognostic factors included age at diagnosis (year), type of surgery (Modified radical mastectomy (MRM) and Breast conserving surgery (BCS), tumor grade (grade 1–3), and stage of disease (Stage 1–4) based on the seventh edition of the TNM classification. The p-values less than 0.05 were considered to be significant. The analysis was performed using STATAversion 12 and quantreg package in R.

### Models

Quantile regression is a statistical technique intended to inference about conditional quantile functions. This method offer a mechanism for estimating models for the conditional median function, and the full range of other conditional quantile functions.

Let T denote the failure time and $\tilde{X }={({X}_{1},{X}_{2},\dots ,{X}_{p})}^{\mathrm{T}}$, denote a p × 1 vector of covariates. let Q_Y_($\uptheta$|$\tilde{X }$) = inf{t: Pr(Y ≤ t|$\tilde{X }$) ≥ $\uptheta$}, denote the $\uptheta$th conditional quantiles of Y = log(T), (or another monotone transformation of T) given $\tilde{X }$, where $\uptheta \in (\mathrm{0,1})$. For randomly censored data, let C denote time to censoring and let $\tilde{{\mathrm T}}=\mathrm{min}\left(\mathrm{T},\mathrm{C}\right)\mathrm{and \delta }=\mathrm{I}\left(\mathrm{T}\le \mathrm{C}\right)$. The observed data consist of n i.i.d replicates of ($\tilde{{\mathrm T}},\updelta ,\mathbf{X})$, denoted by ($\tilde{{\mathrm T}}_{i},{\updelta }_{i},{\mathbf{X}}_{{\varvec{i}}})$, i = 1,…, n. Define $\tilde{{\mathrm Y}}=\mathrm{log}(\tilde{T })$, $\tilde{{\mathrm Y}}_{\mathrm{i}}=\mathrm{log}(\tilde{\mathrm{T}}_{\mathrm{i}})$.

The linear QR model takes the form

1 $${\mathrm{Q}}_{\mathrm{Y}}\left(\uptheta |\mathbf{X}\right)={\mathbf{X}}^{\mathrm{T}}\upbeta \left(\uptheta \right),\uptheta \in \left({\theta }_{L},{\theta }_{U}\right)$$

where $0<{\theta }_{L}<{\theta }_{U}<1, \mathrm{and}$ $\upbeta \left(\uptheta \right)$ is a vector of unknown regression coefficients that represents the change in the $\uptheta$th conditional quantile of Y given a one-unit change in the corresponding covariate^30^. When ${\theta }_{L}$ = ${\theta }_{U}$, model (1) is referred to as a locally linear quantile regression model. When ${\theta }_{L}$ < ${\theta }_{U}$, model (1) referred to as a globally linear quantile regression model.

The most applying methods during the recent years are Portnoy^5^, Wang and Wang^7^, Bottai and Zhang^8^, Yang et al.^9^ and De Backer et al.^10^ methods. In the following, we present a brief overview of methodological framework for these models.

### Portnoy method

Portnoy^5^, using Efron’s^31^ interpretation of Kaplan–Meier as shifting mass of censored observations to the right, proposed an estimation algorithm under the standard random right censoring assumption to estimate $\upbeta \left(\uptheta \right)$.

The grid-based procedure presented in Neocleous et al.^32^ defines a grid of $\uptheta$ -values, ${\mathrm{g}}_{n}$, as, for $0<{\uptheta }_{1}<{\uptheta }_{2}<\dots <{\uptheta }_{\mathrm{K}}={\uptheta }_{\mathrm{U}})$. Let $\Vert {\mathrm{g}}_{n}\Vert =\mathrm{max}\left\{{\uptheta }_{\mathrm{r}}-{\uptheta }_{\mathrm{r}-1}: \mathrm{r}=1, 2,\dots ,\mathrm{R}\right\}$. We will adopt the grid ${\mathrm{g}}_{n}$ throughout this section. $\upbeta \left({\uptheta }_{1}\right)$ is estimated from applying uncensored QR when no censoring occurs below the ${\uptheta }_{1}$th conditional quantile of T. Then, $\upbeta \left({\uptheta }_{\mathrm{r}+1}\right)$ is a value of **b** (**b** is a vector of unknown regression coefficients) minimizing sequentially for r = 1, 2,…, R, by

2 $${\mathrm{min}}\left\{\sum_{{\mathrm{i}}\notin {\mathrm{G}}}{\uprho }_{\uptheta }\left(\tilde{\mathrm{Y}}_{\mathrm{i}}-{\mathbf{X}}_{\mathrm{i}}^{\mathrm{T}}\mathbf{b}\right)+\sum_{\mathrm{i}\in {\mathrm{G}}}{[{\mathrm{w}}_{{\mathrm{r}}+1,{\mathrm{i}}}\uprho }_{\uptheta }\left(\tilde{\mathrm{Y}}_{\mathrm{i}}-{\mathbf{X}}_{\mathbf{i}}^{\mathrm{T}}\mathbf{b}\right)+(1-{\mathrm{w}}_{\mathrm{r}+1,{\mathrm{i}}}){\uprho }_{\uptheta }\left({\mathrm{Y}}^{*}-{\mathbf{X}}_{\mathrm{i}}^{\mathrm{T}}\mathbf{b}\right)]\right\}$$

where ${\mathrm{Y}}^{*}$ is an extremely large value, ${\uprho }_{\uptheta }\left(x\right)=x\left\{\theta -I\left(x<0\right)\right\},$ and $\mathrm{G}$ is the set of indices of censored observations that have been previously crossed. The weight ${\mathrm{w}}_{\mathrm{r}+1,\mathrm{i}}=\left({\uptheta }_{\mathrm{r}+1}-{\uptheta }_{\mathrm{l}}\right)/\left(1-{\uptheta }_{\mathrm{l}}\right)$, approximates the conditional probability $\mathrm{Pr}\left({\mathrm{C}}_{\mathrm{i}}<{\mathrm{T}}_{\mathrm{i}}<\mathrm{exp}\left\{{\mathbf{X}}_{\mathrm{i}}\upbeta ({\uptheta }_{\mathrm{r}+1})\right\}|{\mathrm{C}}_{\mathrm{i}}<{\mathrm{T}}_{\mathrm{i}},{\mathbf{X}}_{\mathrm{i}}\right)$, based on the estimates for $\upbeta \left({\uptheta }_{1}\right),\upbeta \left({\uptheta }_{2}\right)\dots ,\upbeta ({\uptheta }_{\mathrm{r}})$^5^.

### Bottai and Zhang method

Bottai and Zhang^8^, to estimator $\upbeta (\uptheta )$ considered a regression model where the error term is assumed to follow asymmetric Laplace distribution. They explored its use in the estimation of conditional quantiles of a continuous outcome variable given a set of covariates in the presence of random censoring.

They supposed that exists a fixed r-dimensional parameter vector $\upbeta \left(\uptheta \right)$ such that

3 $${\mathrm{T}}_{\mathrm{i}}={\mathbf{X}}^{\mathrm{T}}\upbeta \left(\uptheta \right)+{\upvarepsilon }_{\mathrm{i}}$$

where ${\upvarepsilon }_{\mathrm{i}}$ is an independent and identically distributed residual whose $\uptheta$th quantile equals zero ($\mathrm{P}\left({\upvarepsilon }_{\mathrm{i}}\le 0|{\mathrm{x}}_{\mathrm{i}}\right)=\uptheta ).$

Let ${\mathrm{T}}_{\mathrm{i}}$ conditionally on ${\mathrm{X}}_{\mathrm{i}}$, follows a form of asymmetric Laplace distribution with probability density function

$$\mathrm{f}\left({\mathrm{T}}_{\mathrm{i}}\right)=\mathrm{exp}\left\{\left(\mathrm{I}\left({\mathrm{t}}_{\mathrm{i}}\le {\upmu }_{\mathrm{i}}\right)-\uptheta \right)\frac{{\mathrm{t}}_{\mathrm{i}}-{\upmu }_{\mathrm{i}}}{\upsigma \left(\uptheta \right)}\right\}\frac{\uptheta \left(1-\uptheta \right)}{\upsigma \left(\uptheta \right)}$$

where $$\upsigma \left(\uptheta \right)\in (0,\infty )$$, $${\upmu }_{\mathrm{i}}={\mathbf{X}}^{\mathrm{T}}\upbeta \left(\uptheta \right)$$.

In the presence of censored observations, the likelihood function is proportional to

$$\mathrm{l}\left(\upbeta \left(\uptheta \right),\upsigma \left(\uptheta \right)|{\mathrm{T}}_{\mathrm{i}}\right)=\prod_{\mathrm{i}=1}^{\mathrm{N}}\left({\left(\mathrm{f}({\mathrm{T}}_{\mathrm{i}})\right)}^{{\updelta }_{\mathrm{i}}}{\left(1-\mathrm{F}({\mathrm{T}}_{\mathrm{i}})\right)}^{\left(1-{\updelta }_{\mathrm{i}}\right)}\right)$$

The maximum likelihood estimators for the parameters are defined as maximizes of $\mathrm{l}\left(\upbeta \left(\uptheta \right),\upsigma \left(\uptheta \right)|{\mathrm{T}}_{\mathrm{i}}\right)$. They used algorithm proposed by Nelder and Mead^33^, to estimate parameters and inference on the parameters obtained by bootstrapping the point estimates for quantile of interest^8^.

### Wang and Wang method

A locally weighted method was proposed by Wang and Wang^7^ to estimate a locally linear quantile regression model, which assumes that ${\theta }_{L}$ = ${\theta }_{U}$, in model (1), i.e.

4 $${\mathrm{Q}}_{\mathrm{Y}}\left(\uptheta |\mathbf{X}\right)={\mathbf{X}}^{\mathrm{T}}\upbeta \left(\uptheta \right)$$

Wang and Wang^7^, for random censoring, by twisting the idea of the self-consistent Kaplan–Meier estimator^31^, proposed to modify the standard quantile loss function. The fundamental idea of Wang and Wang^7^ is to redistribute the probability mass $\left(\mathrm{Pr}({\mathrm{T}}_{i}>{\mathrm{C}}_{i}|{\mathrm{C}}_{i},{\mathbf{X}}_{{\varvec{i}}})\right)$, of the censored cases to the right through a local weighting scheme^7^. An estimator of $\upbeta \left(\uptheta \right)$ can be obtained by minimizing the following objective function of β:

5 $${n}^{-1}\sum_{i=1}^{n}[{w}_{i}({F}_{0}){\uprho }_{\uptheta }\left(\tilde{\mathrm{Y}}_{i}-{\mathbf{X}}_{\mathrm{i}}^{\mathrm{T}}\mathbf{b}\right)+\{1-{w}_{i}\left({F}_{0}\right)\}{\uprho }_{\uptheta }\left({\mathrm{Y}}^{*}-{\mathbf{X}}_{\mathrm{i}}^{\mathrm{T}}\mathbf{b}\right)]$$

where ${F}_{0}(t|{\varvec{x}})\equiv \mathrm{Pr}(T>t|{\varvec{X}}={\varvec{x}})$ is known and

$$w_{i} \left( {F_{0} } \right) = \left\{ {\begin{array}{*{20}l} 1 \hfill & {F_{0} \left( {C_{i} {|}{\varvec{X}}_{{\varvec{i}}} } \right) > \theta \;{\text{or}}\; \delta = 1 } \hfill \\ {\frac{{\theta - F_{0} (C_{i} |{\varvec{X}}_{{\varvec{i}}} )}}{{1 - F_{0} (C_{i} |{\varvec{X}}_{{\varvec{i}}} )}}} \hfill & {F_{0} \left( {C_{i} {|}{\varvec{X}}_{{\varvec{i}}} } \right) < \theta \;{\text{and}} \;\delta = 0 } \hfill \\ \end{array} } \right.$$

Wang and Wang^7^ proposed to minimize the objective function (5), when ${F}_{0}\left(t|{\varvec{x}}\right)$ is unknown, with ${F}_{0}()$ replaced by the Beran’s local Kaplan–Meier estimator^34^, $\widehat{F}(.)$,

$$\widehat{F}\left(t|{\varvec{x}}\right)=1-\prod_{j=1}^{n} \Bigg \{1-{\frac{{B}_{nj}(x)}{{\sum }_{k=1}^{n}I({\tilde{Y }}_{k}>{\tilde{Y }}_{j}){B}_{nk}(x)} \Bigg \}}^{{N}_{j}(t)}$$

where $N\left(t\right)=I\left(\tilde{Y }\le t, \delta =1\right),\mathrm{ and}$ ${B}_{nk}(x)$ is a sequence of nonnegative weights adding up to 1, for example, Nadaraya Watson’s type weight, ${B}_{nk}\left(x\right)=K(\frac{x-{x}_{k}}{{h}_{n}})/\sum_{i=1}^{n}K(\frac{x-{x}_{i}}{{h}_{n}})$, where $K$() is a density kernel function and ${h}_{n}$ is a positive bandwidth converging to 0 as n → ∞^7^.

### De Backer method

De Backer et al.^10^ proposed to estimate model (4) based on a minimum distance loss function, given by ${\sum_{i=1}^{n}\{1-\widehat{F}\left({{X}_{i}}^{\uptau }\beta (\theta )|{X}_{i}\right)-\theta \}}^{2}$. They further suggested using a smooth double kernel version of $\widehat{F}\left(.|{X}_{i}\right)$. Let ${Y}_{i}^{u}$ denote the i-th order statistic of the uncensored responses, ${n}^{u}=\sum_{i=1}^{n}{\delta }_{i}$, and let ${H}^{*}\left(t\right)={\int }_{-\infty }^{t}K\left(u\right)du$, for some kernel density $K$. They propose to estimate $F\left(t|{\varvec{x}}\right)$ by ${\widehat{F}}^{s}\left(t|{\varvec{x}}\right)$, where.

$${\widehat{F}}^{s}\left(t|{\varvec{x}}\right)=\int {\varvec{H}}\left(\frac{{\varvec{t}}-{\varvec{u}}}{{{\varvec{h}}}_{{\varvec{T}}}}\right){\varvec{d}}\widehat{F}\left(u|{\varvec{x}}\right)=\sum_{{\varvec{i}}=1}^{{n}^{u}}\left(\widehat{F}\left({Y}_{(i)}^{u}|{\varvec{x}}\right)-\widehat{F}\left({Y}_{(i-1)}^{u}|{\varvec{x}}\right){\varvec{H}}\left(\frac{{\varvec{t}}-{Y}_{(i)}^{u}}{{{\varvec{h}}}_{{\varvec{T}}}}\right)\right)$$

### Yang method

Yang et al.^9^ proposed a new and unified approach, to estimate the quantile regression model (1) with ${\theta }_{U}=1.$ They used a variation of the data augmentation algorithm base on the general principle of data augmentation^15^. The algorithm starts with a set of initial values, ${\widehat{\beta }}^{(0)}({\theta }_{k}), k=1,\dots ,{M}_{n}$, obtained by parallel quantile regression estimators or existing quantile regression estimators. Draw ${Y}_{i}^{(u)},$ for $u=1,\dots ,U$, from the quantile process approximated by ${\mathbf{X}}_{\mathrm{i}}^{\mathrm{T}} {\widehat{\beta }}^{\left(u-1\right)}\left({\theta }_{k}\right),$ conditional on the set of possible values for ${Y}_{i}$. Then, obtain updated estimates ${\widehat{\beta }}^{(u)}\left({\theta }_{k}\right),\mathrm{ via standard uncensored quantile regression},$ based on a pairwise bootstrapping sample of size n from ${\{{\mathbf{X}}_{{\varvec{i}}},{Y}_{i}^{(u)}\}}_{i=1}^{n}$. The final estimates obtain from $\widehat{\beta }\left(\theta \right)={U}^{-1}\sum_{u=1}^{U}{\widehat{\beta }}^{(u)}\left({\theta }_{k}\right)$^9^.

The proposed method adapts easily to different forms of censoring including doubly censored and interval censored data^9^.

### Ethics approval and consent to participate

This study was approved by the Ethics Committee of School of Public Health & Allied Medical Sciences-Tehran University of Medical Sciences. Approval ID: IR.TUMS.SPH.REC.1397.212.

Written informed consent for publication of their clinical details was obtained from the patient relative of the patient. A copy of the consent form is available for review by the Editor of this journal.

## Results

The median follow-up time was 22.32 months with inter-quartile ranged from 13.10 to 30.51 months. During the follow-up, 143 (17.9%) patients died due to breast cancer and 657 (82.1%) survived or censored. Mean (SD) of age at diagnosis was 48.86 (13.63) years, and 106 (13.3%), 586 (73.3%), 65 (8.1%) and 43 (5.4%) patients were diagnosed in stages of disease 1 to 4, respectively. Further, 487 (60.9%) had tumor grade 2, 21 (27.3%) had tumor grade 3. 67 (84.3%) of patients had undergone MRM surgery. The proportional hazard assumption was confirmed at the significant level 0.05.

Table 2 displays the results of analysis of Cox and AFT models. Based on the results of Cox model, tumor grade and stage of disease had a significant effect on breast cancer survival. In other words, the hazard of death was 3.12 times for a higher stage of the disease, while the hazard ratio of death equaled to 1.71 for grade of tumor. In the AFT model with Weibull distribution, a shape parameter equaled to 1.24 that was significantly different from 1. Based on AFT model, the stage of disease and grade of tumor were the factors affecting survival time. Thus, increasing the stage of disease and the grade of tumor, decreased the median (or other quantiles) of survival time by a factor of 0.38 ($\mathrm{exp}(-0.94)$) and a factor of 0.63 ($\mathrm{exp}(-0.45)$) respectively.

**Table 2:** Multivariate analysis of prognostic factors of breast cancer survival with Cox model and AFT model.

Variables	Cox model			AFT model		
	HR	p-value	95% Cl	Coef	p-value	95% Cl
Age	0.99	0.753	(0.98, 1.01)	0.002	0.749	(− 0.01, 0.01)
**Surgical procedure**						
MRM	1.00			1.00		
BCS	0.58	0.079	(0.32, 1.06)	0.40	0.104	(− 0.08, 0.88)
Stage	3.12	< 0.001	(2.55, 3.81)	− 0.94	< 0.001	(− 1.11, − 0.77)
Grade	1.71	< 0.001	(1.27, 2.30)	− 0.45	< 0.001	(− 0.69, − 0.21)

*HR* Hazard Ratio, *Coef.* Estimated parameter, *CI* Confidence Interval, *AFT* Accelerated failure time.

**Figure 1:**
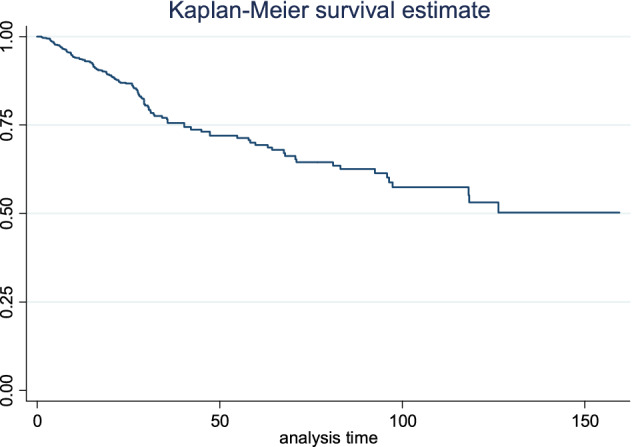
Kaplan–Meier plot of survival time of patient with breast cancer.

As shown in the Kaplan–Meier plot in Fig. 1, at the end of the follow-up the minimum percentile of survival was 51%. Thus, we considered the10th, 20th and 40th percentiles of survival time in CQR model and considered the bandwidth 0.05 that was used in Wang and Wang and De Backer methods. According to the results of all quantile regression models in 20th percentile, tumor grade and stage of the disease were identified as significant factors affecting the survival time. However, the effectiveness of those factors varies in each model. In this regard, in Bottai and Zhang method 20th percentile of survival time for a case with higher unit of stage decreased 14.28 months and for a case with higher grade decreased12.53 months. In Yang method it was 16.64 and 14.53 months less and in Portnoy method 18.06 and 18.31 months, respectively. The result for 10th, 20th and 40th percentiles of survival time were shown in Table 3.

**Table 3:** Multivariate analysis of prognostic factors of breast cancer survival with Portnoy, Wang and Wang, Bottai and Zhang, Yang and De Backer methods.

Quantiles	Coef. (95% Cl)		
	0.10	0.20	0.40
**Bottai and Zhang method**			
Intercept	48.96 (22.65, 75.26)*	79.97 (44.82, 115.12)*	133.74 (87.57, 179.92)*
Age	0.02 (− 0.23, 0.26)	0.03 (− 0.30, 0.36)	0.09 (− 0.36, 0.53)
Surgical procedure (BCS)	11.28 (− 2.97, 25.54)	14.34 (− 1.07, 29.75)	24.07 (3.03, 45.14)*
Stage	− 10.59 (− 14.23, − 6.95)*	− 14.28 (− 19.01, − 9.56)*	− 28.87 (− 35.001, − 22.74)*
Grade	− 6.85 (− 13.14, − 0.57)*	− 12.53 (− 20.88, − 4.18)*	− 12.39 (− 23.60, − 1.19)*
**Portnoy method**			
Intercept	56.94 (37.86,76.65)*	102.05 (81.86,145.50)*	199.77 (87.30,304.03)*
Age	0.08 (− 0.23,0.37)	0.19 (− 0.99,0.62)	0.06 (− 0.60,0.45)
Surgical procedure (BCS)	14.34 (5.49,27.91)*	10.36 (1.35,22.13)*	22.31 (− 26.34,116.88)
Stage	− 12.61 (− 16.96, − 8.56)*	− 18.06 (− 24.26, − 6.85)*	− 43.58 (− 49.06, − 39.17)*
Grade	− 10.24 (− 13.48, − 3.36)*	− 18.31(− 25.81,13.60)*	− 13.92 (− 32.98, 20.41)
**Wang and Wang method**			
Intercept	17.57 (0.51, 45.50)*	42.05 (5.51, 69.10)*	111.39 (34.85, 160.72)*
Age	0.02 (− 0.19, 0.19)	− 0.02 (− 0.22, 0.25)	− 0.08 (− 0.48, 0.56)
Surgical procedure (BCS)	11.95 (3.42, 31.14)*	17.68 (− 1.85, 35.57)	8.96 (− 8.36, 60.73)
Stage	− 5.26 (− 8.41, − 2.69)*	− 8.26 (− 11.37, − 5.19)*	− 18.59 (− 24.51, − 10.65)*
Grade	− 2.33 (− 8.11, 0.32)	− 6.51 (− 10.14, − 0.45)*	− 11.57 (− 25.53, − 3.30)*
**De Backer method**			
Intercept	52.79 (25.79, 82.24)*	73.91 (40.12, 126.08)*	123.42 (74.45, 171.28)*
Age	0.02 (− 0.31, 0.24)	− 0.07 (− 0.36, 0.41)	− 0.03 (− 0.46, 0.58)
Surgical procedure (BCS)	3.62 (− 5.37, 25.95)	9.24 (− 5.37, 27.11)	10.98 (− 15.25, 41.63)
Stage	− 10.05 (− 13.62, − 5.88)*	− 12.56 (− 20.15, − 9.09)*	− 26.81 (− 35.98, − 15.86)*
Grade	− 5.91 (− 14.88, − 0.81)*	− 8.76 (− 19.49, − 3.01)*	− 5.30 (− 21.46, 2.24)
**Yang method**			
Intercept	53.11 (23.09, 93.38)*	87.20 (44.17, 133.21)*	198.89 (44.49, 227.96)*
Age	0.06 (− 0.26, 0.32)	0.12 (− 0.23, 0.51)	0.03 (− 0.29, 0.49)
Surgical procedure (BCS)	15.63 (− 1.59, 31.70)	14.71 (− 0.92, 37.20)	11.05 (− 16.74, 30.51
Stage	− 12.04 (− 17.59, − 7.07)*	− 16.64 (− 23.77, − 9.74)*	− 40.23 (− 41.28, − 9.74)*
Grade	− 9.68 (− 16.89, − 2.70)*	− 14.53 (− 26.03, − 5.47)*	− 14.20 (− 25.80, − 3.51)*

*Coef.* Estimated parameter, *CI* Confidence Interval.

*P-value < 0.05.

**Figure 2:**
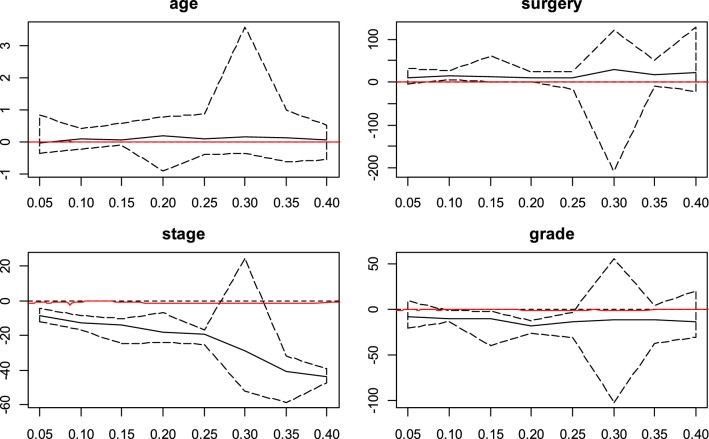
Censored quantile regression coefficients plots and their confidence intervals (dashed line) for Portnoy method and conditional quantile effects estimated by Cox model (red line).

**Figure 3:**
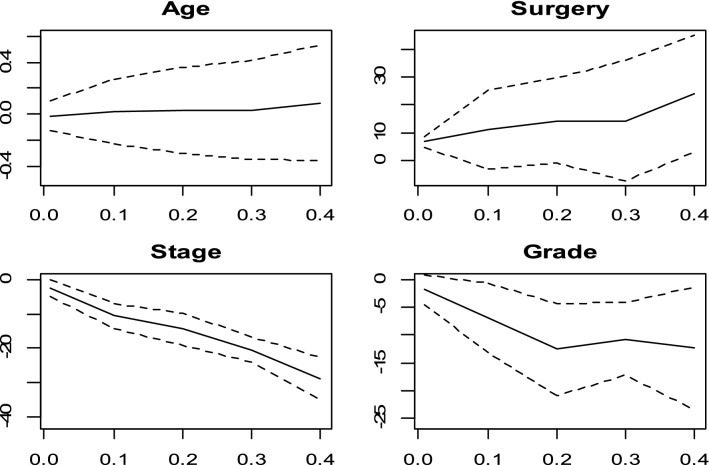
Censored quantile regression coefficients plots and their confidence intervals (dashed line) for Bottai and Zhang method.

**Figure 4:**
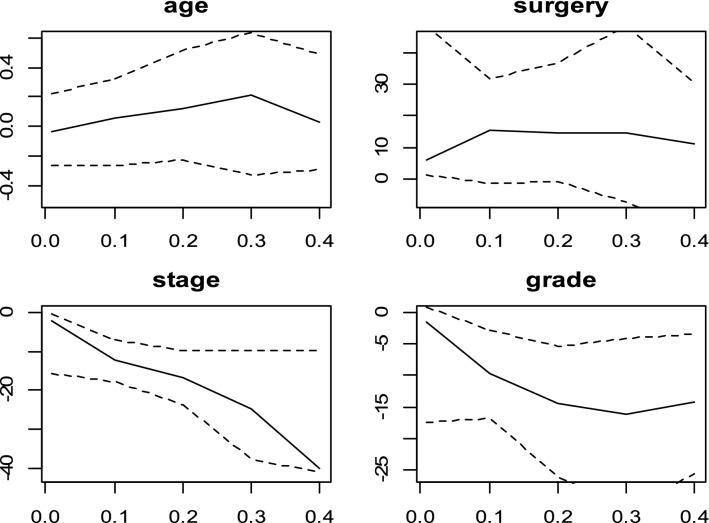
Censored quantile regression coefficients plots and their confidence intervals (dashed line) for Yang method.

**Figure 5:**
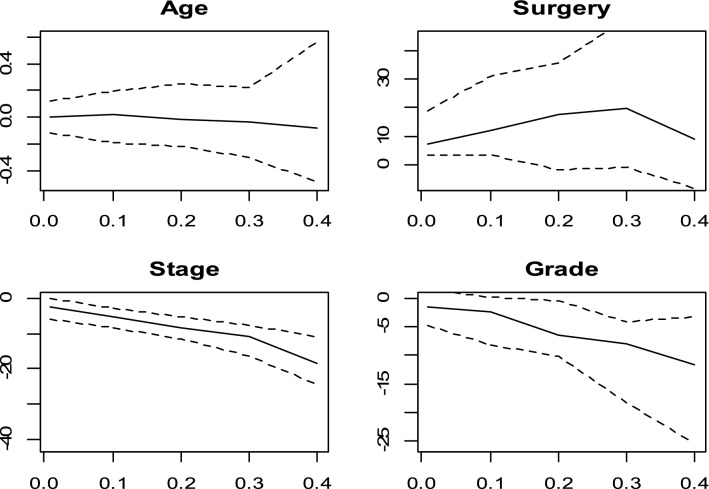
Censored quantile regression coefficients plots and their confidence intervals (dashed line) for Wang and Wang method.

**Figure 6:**
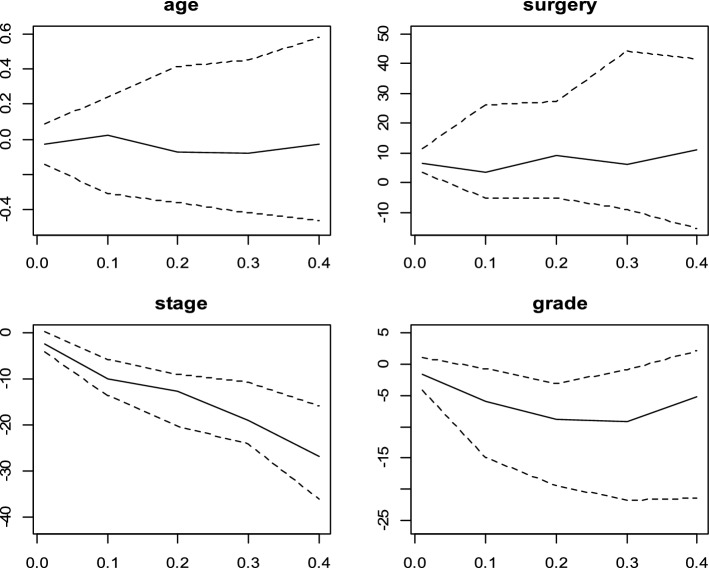
Censored quantile regression coefficients plots and their confidence intervals (dashed line) for De Backer method.

**Figure 7:**
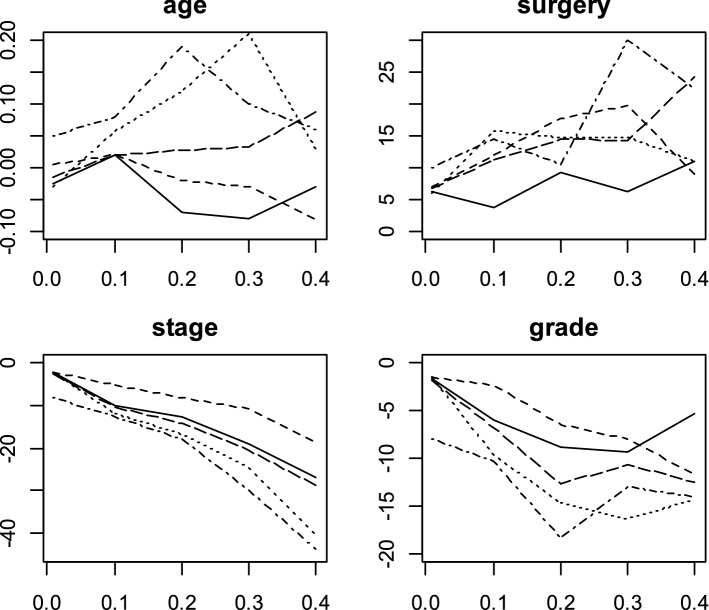
Censored quantile regression coefficients plots for Portnoy (dotdash line), Wang and Wang (dashed line), Bottai and Zhang (longdash line), Yang (dotted line), and De Backer (solid line) methods.

The CQR coefficients estimated and the 95% confidence intervals (CI) with Portnoy, Bottai and Zhang, Yang, Wang and Wang and De Backer methods and conditional quantile effects estimated by Cox model for $\uptheta$ ∈ (0.01, 0.10, …, 0.40) were displayed in Figs. 2, 3,4,5 and 6. Figure 7 shows the estimated coefficients of five methods. In the Cox model, the estimated quantile measure for each covariate was computed using Eq. (9) of Portnoy^5^. The effects of the Cox model were almost the same in different quantiles while they changed in quantile regression models as the quantiles vary.

## Discussion

In the present study, CQR methods were compared in modeling the prognosis factors of breast cancer survival based on the breast cancer data collected from Imam Khomeini Hospital. Tehran, Iran.

The analyses of accelerated failure-time models and Cox model showed that the stage of disease and grade of tumor were identified as the prognostic factors of breast cancer survival. According to the Cox model, the stage of disease and tumor grade increased the risk of death. In the AFT model, the median of survival time decreased as the stage of disease and tumor grade increased. The analyses of CQR models showed that type of surgery, stage and grade were the effective factors on the survival of patients with breast cancer. These results are consistent with most findings in the existing literature, although a direct comparison of the effect size is difficult due to the fact that the majority of works report hazard ratios or odds ratios. Among the significant factors in this study, the effect of surgical method on the survival of breast cancer patients is one of the most addressed issues in recent studies^35–37^. Our study showed that 20^th^ percentile survival time increased for women with BCS surgery, based on Portnoy methods. In the study of Hofvind, by controlling other factors, the hazard of death in MRM is 1.7 times higher than BCS^38^. Meanwhile, there has been no significant difference between two surgical methods in the study of Quan^39^. In most of quantiles stage and grade have significant effect on survival. In recent study, Saadatmand describe overall survival of female patients with breast cancer from two time cohorts (1999–2005 and 2006–2012) in a nationwide population based study^40^. Their results emphasize the importance of tumor stage at diagnosis of breast cancer, as it still greatly affects overall survival. Rottenberg examine differences in survival among older women diagnosed with breast cancer, according to age and disease stage at time of diagnosis. And showed that stage disease among older women became a less powerful predictor of mortality with rising age^41^.

The CQR models allow covariate effects to change in people with different risk. Thus, it is a flexible model for controlling heterogeneity due to covariates. In our studies, coefficients of prognostic factor were different in quantiles that showed different effect of prognostic factor survival time in each quantile. Base on Portnoy method high stage of disease decreased 10th, 20th and 40th percentiles survival time 12.61, 18.06 and 43.58 months, respectively.

The difference in the interpretation of the parameters is the biggest one among these models. The Cox model examines the covariate effects on the hazard function. In addition, the Cox model shows the hazard of death per unit of increase in covariate when the event is death. In this study, the hazard of death was 3.12 times for a higher stage of the disease, and the hazard of death was 1.71 times for a higher degree of tumor grade. Contrary to the proportional hazards model which describes how predictors influence the hazard function, the AFT model assumes a direct association between predictors and survival time, which makes interpretation easier. If the median survival time to event is considered and the accelerate factor is greater than one, the median survival time increases by the accelerate factor with increasing one unit covariate while the median survival time to event decreases if it is less than one. In the present study, based on AFT model, the median survival time decreased by a factor of 0.38 and 0.63 with increasing stage of disease and grade of tumor, respectively. The results of this model can be expressed as a proportional hazard, in which the interpretation is similar to the Cox model. The interpretation of coefficients in CQR model is considered as the changing rate quantile of dependent variable per one unit change in independent variable, like other linear models. If the event is death, it can be expressed as the covariate effects on the patients’ lifetime.

Modeling the breast cancer data with CQR models indicated that most of the time all models acted the same to determine prognostic factors of breast cancer survival but sometime, significant factors and their coefficients were different. All models considered the stage of disease and grade of tumor as prognosis factors. With regard to the coefficients of covariates in different quantiles, the coefficients of Portnoy and Yang method were close to each other and the coefficients of Bottai and Zhang and De Backer methods were close to each other and they were different from Wang and Wang method. Peng and Huang compared their method with Portnoy's method and showed that both methods could represent very similar results^6^. Bottai and Zhang compared Laplace regression method with Peng-Huang and Portnoy methods by using simulation and indicated that the advantages of their method include giving the same results and accurate convergence, while two other methods sometimes failed to converge, and involve fast calculations^8^. Wang and Wang showed that the new approach adopts a preliminary local Kaplan–Meier estimator and results a weighted quantile regression. They established, utilizing results in modern empirical process theory, the consistency and asymptotic normality of the resulted estimator^7^. Base on simulation studies and the analysis of real data, the proposed method has shorter interval estimates than Portnoy’s procedure^7^. De backer et al. in their study indicated in an extensive simulation study that the resulted quantile regression estimator respect to established check-based formulations have less variance results. From a theoretical prospect, both consistency and asymptotic normality of the proposed estimator for linear regression are obtained under classical regularity conditions^10^. Yang et al. indicated that the Yang's method presents an estimator is able to achieve significant efficiency gains in comparisons with Portnoy’s estimator^9^.

The assumptions required in each of the models should be considered while using these models. The proportional hazards assumption is the most important assumption of the Cox model and what it means is that the ratio of the hazards for any two individuals is constant over time. In this model, no assumptions are made about form of the baseline hazard. However, the distribution of survival time is sometimes specific or assuming a parametric form is logical. In these cases, parametric methods are used. Common parametric distributions in survival models include Weibull, Generalized Gamma, Log-Normal, Log-Logistic. In Bottai and Zhang method, it is assumed that the error terms follow asymmetric Laplace distribution. However, Yu and Moyeed (2001) showed that the model performs well when the error terms follow other distributions^42^. Portnoy and Yang methods require just global linearity assumption^5,9^. Wang and Wang and De Backer methods have local linearity assumption^7,10^.

Computational time is another important issue in comparing these models. According to our data, the computational time of Portnoy, Bottai and Zhang and Yang methods is shorter than other methods.

It is necessary task to measure the goodness of survival models. Although for the model diagnostics of quantile regression with complete data some tools, such as the worm plot, have been proposed, for censored quantile regression is still greatly underdeveloped^43,44^. Designing effective model diagnostic tools for censored quantile regression warrants more in-depth research.

The high percentage of right censoring is regarded as one of the limitations of this study. By this way, modeling 50th percentile of survival time requires more follow-up time to increase the percentage of event. Thus, the comparison of models in higher quantiles was not possible.

## Conclusions

For the CQR models, various approaches have been proposed that the most practical of them include: Portnoy^5^, Wang and Wang^7^, Bottai and Zhang^8^, Yang et al.^9^ and De Backer et al.^10^ methods. Portnoy^5^ generalized the Kaplan–Meier method with a recursively weighted estimation algorithm under the global linearity assumption of the conditional quantile functions. To overcome the linearity assumption, Wang and Wang^7^ developed a method by non-parametrically estimating the conditional survival distribution via kernel smoothing. In 2010, Laplace regression was introduced as a parametric method for modeling the conditional quantile of censored data by Bottai and Zhang. Yang et al.^9^ employed a variation of the data augmentation algorithm base on the general principle of data augmentation^15^. De Backer et al.^10^ investigate a new procedure that used “check” loss function. The CQR methods acted the same to determine prognostic factors of breast cancer survival in most of the time. The estimated coefficients of five methods were close to each other for quantiles lower than 0.1 and they were different from quantiles upper than 0.1.

## Data Availability

The datasets used and/or analyzed during the current study are available from the corresponding author on reasonable request.

## Abbreviations

AFT: Accelerated failure time
BCS: Breast conserving surgery
MRM: Modified radical mastectomy
QR: Quantile regression
